# Characterization of Ag-Ion Releasing Zeolite Filled 3D Printed Resins

**DOI:** 10.3390/jfb14010007

**Published:** 2022-12-22

**Authors:** Marian O. Pacho, Dylan Deeney, Emily A. Johnson, Bryanna N. Bravo, Kishen Patel, Mark A. Latta, Michael A. Belshan, Stephen M. Gross

**Affiliations:** 1 Department of Oral Biology, School of Dentistry, Creighton University, Omaha, NE 68178, USA; 2 Department of Medical Microbiology and Immunology, Creighton University, Omaha, NE 68178, USA; 3 Department of Chemistry, College of Arts and Sciences, Creighton University, Omaha, NE 68178, USA

**Keywords:** 3D printing, zeolites, antiviral, dental resin, SARS-CoV2, silver

## Abstract

There has been profound growth in the use of 3D printed materials in dentistry in general, including orthodontics. The opportunity to impart antimicrobial properties to 3D printed parts from existing resins requires the capability of forming a stable colloid incorporating antimicrobial fillers. The objective of this research was to characterize a colloid consisting of a 3D printable resin mixed with Ag-ion releasing zeolites and fumed silica to create 3D printed parts with antiviral properties. The final composite was tested for antiviral properties against SARS-CoV-2 and HIV-1. Antiviral activity was measured in terms of the half-life of SARS-CoV-2 and HIV-1 on the composite surface. The inclusion of the zeolite did not interfere with the kinetics measured on the surface of the ATR crystal. While the depth of cure, measured following ISO4049 guidelines, was reduced from 3.8 mm to 1.4 mm in 5 s, this greatly exceeded the resolution required for 3D printing. The colloid was stable for at least 6 months and the rheological behavior was dependent upon the fumed silica loading. The inclusion of zeolites and fumed silica significantly increased the flexural strength of the composite as measured by a 3 point bend test. The composite released approximately 2500 μg/L of silver ion per gram of composite as determined by potentiometry. There was a significant reduction of the average half-life of SARS-CoV-2 (1.9 fold) and HIV-1 (2.7 fold) on the surface of the composite. The inclusion of Ag-ion releasing zeolites into 3D-printable resin can result in stable colloids that generate composites with improved mechanical properties and antiviral properties.

## 1. Introduction

Dentistry has seen tremendous growth in three-dimensional (3D) printing. Modern-day dental clinics are now encouraged to equip themselves with this technology. Dental labs are showing an increased interest in production through additive manufacturing processes. Numerous reviews have been published regarding 3D printing in dentistry [1,2,3,4]. Applications include but are not limited to orthodontics [5], prosthetic dentistry [6], and regeneration of tooth tissues and tooth-supported tissues [7]. Additive manufacturing (AM) products can be constructed based on CBCT scans to produce surgical guides, custom trays, working casts, and provisional restorations [5]. It is essential to consider the aging of the product, material composition, processing parameters, and post-processing parameters when determining the effectiveness of these products [3].

3D printed composites demonstrated better surface and mechanical properties than bis-acrylic resin to support temporary resins [8]. A study developed antibacterial stereolithography resins with Ag-carrying halloysite nanotubules in digital light projection printers, with proven antibacterial properties [9]. Another study showed that alkyl chains that are directly copolymerized with conventional resin components as a linear chain and then incorporated into semi-interpenetrating polymer networks can result in antibacterial properties [10]. Another study indicated that adding 0.4% TiO_2_ nanoparticles with poly (methyl methacrylate) (PMMA) features insulating properties that are usable in 3D printable technology [11]. A 2020 review has also shown that zirconia, which features antimicrobial characteristics, can be processed to be utilized in AM [12]. 

The incorporation of antimicrobial fillers is necessary to generate durable 3D printed materials alongside the growth of AM processes. Published reviews include topics on antiviral agents for oral viruses [13], antiviral restorative materials [13], antimicrobials in dentistry [14], and antimicrobials in biomaterials and technology [15]. Major categories of antimicrobial components in dentistry include nanoparticles, chlorhexidine, chitosan, hydrogen peroxide, and titanium.

Antimicrobial nanoparticle usage in dentistry is growing, seen by a spike of reviews in the past three years [16,17,18,19,20,21,22,23]. Although this study focused on the potential antiviral behavior of Ag ions, a significant amount of work in the field has focused on the antibacterial properties caused by incorporation of Ag nanoparticles (AgNPs). AgNPs are the most noted nanoparticle with numerous reviews [24,25,26,27,28,29,30,31,32] due to their high antimicrobial activity. AgNPs exemplify effective antibacterial characteristics but do better against Gram-positive anaerobes than Gram-negative anaerobes [33]. Effective antimicrobial activity was found when using incolicidin-coated AgNPs against Gram-positive and Gram-negative bacteria regardless of the amount of AgNPs used [34]. AgNPs have various orthodontic uses [35] and are effective against endodontic and periodontal pathogens [36], Streptococcus mutans [37], and bacteria resulting in dental caries formation [38]. New studies have shown manufactured AgNPs using biological agents, which are more environmentally safe and more cost-effective [39]. Due to its popularity, Ag is added alongside other materials to enhance antimicrobial activity, such as PMMA [40], diammine fluoride [41], graphene oxide [42], vanadium [43], gold and heparin [44], chlorhexidine [45], and flower and seed extracts [46]. 

Another antimicrobial included in dental materials are zeolite fillers capable of releasing silver, copper, or zinc ions. These zeolites present sustained release of ions with additional antimicrobial properties. The study of zeolite incorporation has been studied in endodontics, implantology, prosthetics, and restorative dentistry to provide antimicrobial properties. A current review reported antimicrobial characteristics and high mechanical properties when ion-loaded zeolites were incorporated into non-acrylic prosthetic materials [47]. Lower concentrations of zeolites were recommended for mineral trioxide aggregate and acrylic prosthetic materials since higher concentrations decreased mechanical properties [47]. Ion releasing zeolite antimicrobial properties are highly enhanced when combined with glass ionomer cements, depending on the type of glass ionomer cement utilized [47]. Silver exchanged EMT zeolites have also been studied to be incorporated into dental adhesives [48]. A previous study looked at the effects of human coronavirus strain 229E with sodium aluminosilicate zeolites. It was determined that silver and silver/copper-infused zeolite powders significantly decreased coronavirus 229E after a 1 h suspension [49]. Additionally, the study showed that coronavirus exposure to plastic coupons embedded with silver/copper-infused zeolite showed a significant reduction 24 h compared to the positive control [49].

Additional nanoparticles that provide effective antimicrobial properties include chlorhexidine [50,51,52,53], gold [54], graphene-based nanomaterials [55,56,57], magnesium oxide [58], metal oxide [59], quaternary ammonium [60,61,62,63], selenium [64], and zirconia [65]. Dental cements have implemented antimicrobial components for years [66,67]. Components include calcium phosphate [68], Zinc antimicrobial glass [69], and tetracalcium phosphate cements [70]. Additionally, chitosan [71] and chlorhexidine [72,73] have been placed into dental gels for antimicrobial delivery to the oral cavity. Glass ionomers with antimicrobial activity available in the market are Argion (VOCO, Cuxhaven, Germany), Zirconomer (Shofu Inc., Kyoto, Japan), EQUIA Forte (GC, Tokyo, Japan), Fuji II LC capsule (GC, Tokyo, Japan), and Fuji IX GP capsule (GC, Tokyo, Japan) [74].

Viral pandemics occur with regularity throughout the world. Prominent viral outbreaks in the last century have been caused by influenza A and B, human immunodeficiency virus type I (HIV-1), hepatitis B and C, and more recently members of the coronavirus family, including severe acute respiratory virus 1 and 2 (SARS-Cov-1 and -2, respectively). As obligate intracellular parasites, viruses rely on specific mechanisms to transmit from host to host. During this process, viruses are exposed and vulnerable to inactivation through physical, chemical, and energetic mechanisms. Transmission is thus dependent on environmental factors, including temperature, moisture, pH, humidity, and material exposure. Variations in these factors, natural or artificial, may enhance, diminish, or ablate virus transmission from host to host. With sufficient scaling, alterations in transmission factors are postulated to have the potential to alter the trajectory of epidemics. Heavy metals, including silver (Ag) are among the materials that have been demonstrated to exhibit antiviral properties [75,76,77,78,79,80].

The oral cavity, rich in saliva and the oral microbiome, can host these respiratory viruses and is a high viral replication site [81,82]. Dentists are at high risk compared to other healthcare workers due to multiple aerosol-generating procedures within close proximities of patients [83]. The COVID-19 pandemic has significantly impacted dentistry as a highly contaminable respiratory illness [84,85,86,87]. ACE2 is an enzyme receptor that binds to SARS-CoV, and a majority is found in salivary ducts [88] and epithelial cells of the tongue [89], making the oral cavity a highly susceptible area of transmission and infection for the virus [88,89]. Multitudes of studies have outlined methods to prevent contamination with the use of high-volume evacuation [90,91], intraoral and extraoral suctions [90,92], use of nonsurgical procedures [93,94], mouth rinses [95,96,97], rubber dams [98], and PPE [99]. Although these preventative methods are often practiced in dentistry, adding antimicrobial components may benefit both patients and health care professionals in viral transmissions, such as COVID-19.

In this study we explored the virucidal activity of Ag-ion releasing zeolite fillers incorporated into 3-D printing resin. Two viral pathogens of significant concern were investigated, SARS-CoV-2 and HIV-1. Both are membraned viruses from distinct families with different modes of transmission. SARS-CoV-2 is the etiologic agent of the respiratory disease pandemic COVID-1 [100,101]. It is a member of the β coronavirus family and genetically related to SARS-CoV-1 and SARS-related bat CoVs [102,103]. It is an enveloped, single-stranded, positive sense RNA virus. Its primary mode of transmission is airborne through respiratory droplets or fomites. The virus replicates primarily in the respiratory tract epithelium. HIV-1 is the causative agent of acquired immune deficiency syndrome (AIDS; [104,105]). HIV-1 is an RNA virus and member of the *lentiviridae* subfamily of retroviruses. Members of this family are unique for undergoing reverse transcription during replication and integrating into the host genome. HIV-1 infects CD4 cells of the immune system, specifically CD4 T-cells, macrophages, and dendritic cells. It is primarily transmitted via sexual contact or via blood.

This study aims to expand the capability of developing 3D printed materials with antiviral properties. While there has been a significant number of approaches to creating antimicrobial fillers for use in creating composites with antiviral properties, there is still a dearth of information in the literature related to incorporating these types of fillers into materials that can be printed by common 3D printers (e.g., SLA) from colloids containing fillers with antiviral properties. In this study we aimed to determine the effects of Ag-ion releasing zeolites incorporated into 3D printable materials in dentistry since the need for antiviral filler addition to dental materials has spiked due to SARS-CoV-2. Topics explored include polymerization kinetics, depth of cure, flow colloidal stability, viscosity, flexural strength, and the virucidal activity of Ag-ion releasing zeolite composites that could be used in 3D printing in dentistry or on-demand PPE for the healthcare provider.

## 2. Materials and Methods

### 2.1. Materials Used

The colloids formed in this experiment consisted of Surgical Guide VI resin (Formlabs, Ohio), Ag ion-releasing zeolite (2.5 *w*/*w*% Ag) as an antiviral filler was obtained from Sciessent (Sciessent, Beverly, MA, USA) and Aerosil OX50 fumed silica (Parsippany, NJ, USA).

### 2.2. Formulations and Scanning Electron Microscopy (SEM)

10 *w*/*w*% Ag-ion releasing zeolite was mixed with the surgical guide resin in four equal, but separate increments. This allows for better mixing and a reduction in agglomeration of the fillers. Fumed silica (either 3 *w*/*w*%, 6.5 *w*/*w*% or 10 *w*/*w*%) was then mixed into the resin/zeolite mixture in four separate increments under vacuum. In order to determine that the zeolites were homogeneously dispersed in the resin, images of the composite were obtained on a TM3000 scanning electron microscope (Hitachi, Tokyo, Japan).

### 2.3. Polymerization Kinetics

Infrared spectroscopy was used to determine how the antiviral fillers impacted the polymerization kinetics of the 3D printable resin. A Nicolet Avatar 370 DTGS equipped with an ATR crystal was used to measure the conversion of the resin as a function of time. As a control 0.2 mL of neat, unfilled surgical guide resin was polymerized in situ with a VALO GRAND curing light with a standard power of 1000 mW/cm^2^ (Ultradent, South Jordan, UT, USA) at 5 s intervals for a total of 40 s. A colloid prepared with 10 *w*/*w*% Ag-ion releasing zeolite was polymerized under identical conditions. The peaks with frequencies of ≈1635 cm^−1^ and ≈1704 cm^−1^ were recorded and used in the calculation of conversion.

### 2.4. Depth of Cure

The method outlined in ISO4049 was used to determine the depth of cure of the neat and filled 3D-printable resin. Accordingly, a glass microscope slide was placed on a flat surface. A layer of mylar was placed on top of the glass slide. Next, a 12 mm cylindrical mold was placed on the mylar. The mold was filled with either neat surgical guide resin or the colloid prepared with 10 *w*/*w*% Ag-ion releasing zeolite. The filled mold was subsequently covered with another layer of mylar and topped with a second glass slide. The samples were then cured with a VALO GRAND curing light at a power of 1000 mW/cm^2^.

The colloid was cured for 5, 10 and 40 s. After the cure time, the sample was removed from the mold. The polymerized rod was scraped with a spatula to remove the gelatinous portion. A micrometer was used to measure the lengthwise distance of the remaining structure. The recorded distance was halved and reported as the depth of cure.

### 2.5. Flow and Stability

0.030 g of each formulation was placed on a horizontal glass microscope slide. The glass slide was then held vertically, perpendicular to a flat surface, allowing the colloid to flow. The flow distance was measured after 30 s. Formulations were stored over 6 months. Colloidal stability was tested by monitoring the flow of the formulation at different intervals for 6 months.

### 2.6. Viscosity

Viscosity measurements were determined at 23 °C using a DV3 Brookfield Engineering Rheometer in a small sample chamber using the SC4-18 spindle. Colloids were sheared for a total of 270 s at 3 different speeds (1, 10, and 25 RPM) for 90-s intervals at each speed.

### 2.7. Flexural Strength

20 mm × 2 mm × 2 mm specimens (*n* = 10) were formed using a VALO GRAND curing light at a power of 1000 mW/cm^2^. The samples were stored at 37 °C temperature for 7 days and polished. The samples then submitted to a three-point bend test using an MTS Insight Electromechanical instrument.

### 2.8. Silver Ion Release

The Ag-ion releasing zeolite colloid was transferred from a syringe into 60 standard flat Nylon washers (Washers USA, Black Mountain, NC, USA) that were attached to a total of 20 glass microscope slides. The total surface area of exposed composite was 71.3 mm^2^ with a volume of 57.9 mm^3^. The colloid was cured for 40 s on both the top and bottom side using a VALO GRAND curing light at 1000 mW/cm^2^. A DENTSPLY Triad^®^ Visible Light Cure System was used to cure the samples for 5 additional minutes. The composite containing slides were submerged into 200 mL of ultrapure water acquired from a Thermo Scientific Barnstead filtration system. The slides were placed back to back in a sterilized slide staining specimen holder. Aliquots were taken for measurement once a day for 3 days. Potentiometry (ion specific electrode) was used to determine the concentration of silver ions released from the composite. A Thermoscientific Orion Silver/Sulfide electrode was used for the silver ion concentration measurement.

### 2.9. Cell Culture and Virus Assays

293T and derivatives, TZM-bl, and Vero E6-TMPRSS2-T2A-ACE2 (VeroE6-A2T2) were cultured in Dulbecco’s modified eagle media supplemented with 10% fetal bovine serum (Hyclone, Logan, UT, USA), 8 mM·L-glutamine, 100 U/mL penicillin, and100 U/mL streptomycin (complete DMEM). Cells were cultured in humidified incubators at 37 °C and 5% CO_2_. SARS-CoV-2 virus was isolated from a deidentified COVID-19 positive nasal/pharyngeal sample as described [106]. Virus stocks were produced by infection of Vero E6-A2T2 cells for 72 h. Collected supernatants were clarified, aliquoted and stored at −80 °C. SARS-CoV-2 stocks were titered by plaque assays using the solid overlay method described in [107]. HIV-1 NLX [108] virus stocks were produced by transient transfection of 293T cells using as previously described [109]. HIV-1 was titered by infection of TZM-bl indicator cells essentially as described [110], but performed using 96-well plates with 1.2 × 10^4^ cells/well. Luciferase assays were quantified using a Synergy HTX Multi-Mode reader (Agilent, Santa Clara, CA, USA).

Surface exposure experiments were performed by depositing a 100 µL aliquot of SARS-CoV-2 onto each 3-D printed composite for each timepoint (0, 4, 8, 24, 48, and 72 h). A negative, media only, control sample was included in each experiment. At each timepoint, 50 μL of virus was recovered and diluted 1:10 in complete DMEM (1:10 dilution). Samples were immediately stored at −80 °C. At the end of the time course, each sample was titered on Vero E6-A2T2 cells in triplicate as described above. Titer data over time of exposure was plotted using GraphPad Prism software (version 9.4.1, year 2022), from which one phase exponential decay curves were generated and the half-life of the virus calculated. The data presented shows the average half-life of virus on each surface averaged from at least three independent exposure experiments. The HIV-1 exposure experiments were performed similarly, except the recovered virus was diluted only 1:4 in complete DMEM and each timepoint sample was titered in triplicate using TZM-bl cells as described above. Statistical differences in the average half-lives were analyzed by unpaired *t*-test using Graphpad Prism software (version 9.4.1, year 2022).

## 3. Results

### 3.1. Scanning Electron Microscopy

**Figure 1:**
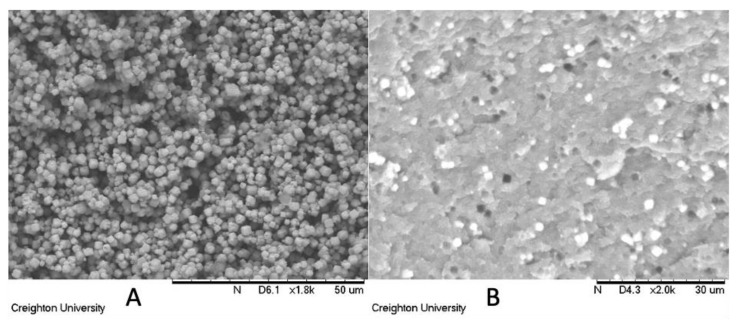
Scanning electron microscopy was used to image the Ag-ion releasing zeolites (**A**) and the composite with the dispersed fillers (**B**).

In order to discern that the particle fillers were homogenously dispersed with nominal agglomeration in the continuous phase, scanning electron microscopy of a sample coupon was obtained. Figure 1A shows the SEM of the neat zeolites. Figure 1B shows the SEM of the polymerized composite.

### 3.2. Polymerization Kinetics

**Figure 2:**
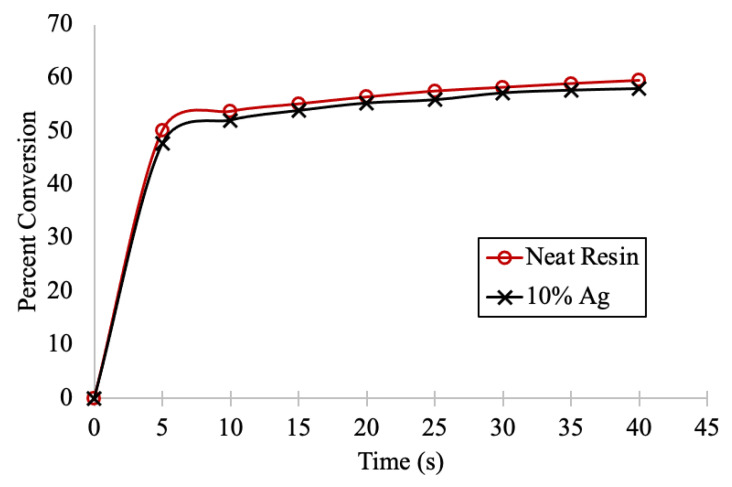
Infrared spectroscopy was used to measure the percent conversion as a function of time (s) for neat resin and the colloid with 10 *w*/*w*% Ag-ion releasing zeolites.

Infrared spectroscopy (FTIR) equipped with an ATR crystal was used to measure the rate of polymerization of the neat resin and was compared to the colloid containing the 10 *w*/*w*% Ag-ion releasing zeolite. The percent conversion of the alkene functional group was measured as a function of time. The absorbance of the alkene monomer at 1635 cm^−1^ was divided by the absorbance of the carbonyl at 1792 cm^−1^ post cure. This ratio was divided by the absorbance ratio of the alkene monomer (1635 cm^−1^) and carbonyl (1792 cm^−1^) pre-cure. The ratio of the absorbances of the polymer divided by the monomer was subtracted from 1 and converted into percent. As seen in Figure 2, both the neat surgical guide resin and the colloid with 10 *w*/*w*% Ag zeolite showed the highest increase in the rate of conversion in the first 5 s of curing. Between 5 and 40 s, a more gradual increase was observed. With a 95% confidence level (Tukey’s method), percent conversion of the neat resin and the zeolite filled colloid were similar.

### 3.3. Depth of Cure

**Figure 3:**
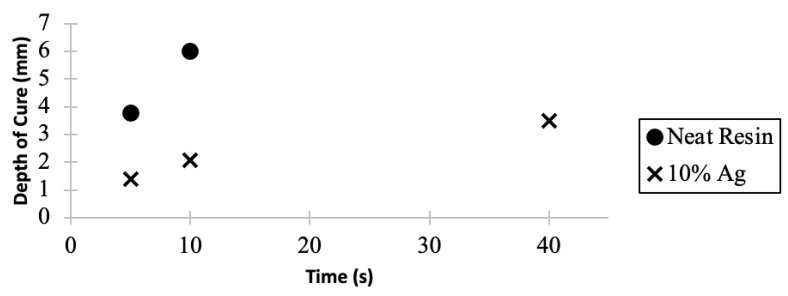
Depth of cure (mm) measurements as a function of time (s) for surgical guide resin and the colloid with 10 *w*/*w*% Ag releasing zeolites.

The depth of cure of neat surgical guide resin and a colloid loaded with 10 *w*/*w*% Ag-ion zeolite is shown in Figure 3. The zeolite loaded colloid showed a decreased depth of cure compared to the neat resin. After eight times the amount of cure time, the 10 *w*/*w*% Ag-ion zeolite loaded resin cured to a comparable depth of cure as the neat resin. The unfilled resin had a depth of cure of 3.8 mm in 5 s. The observed depth of cure for the 10 *w*/*w*% Ag zeolite loaded resin was 1.4 mm after 5 s, and was 3.8 mm after 40 s.

### 3.4. Colloid Flow Measurement

**Figure 4:**
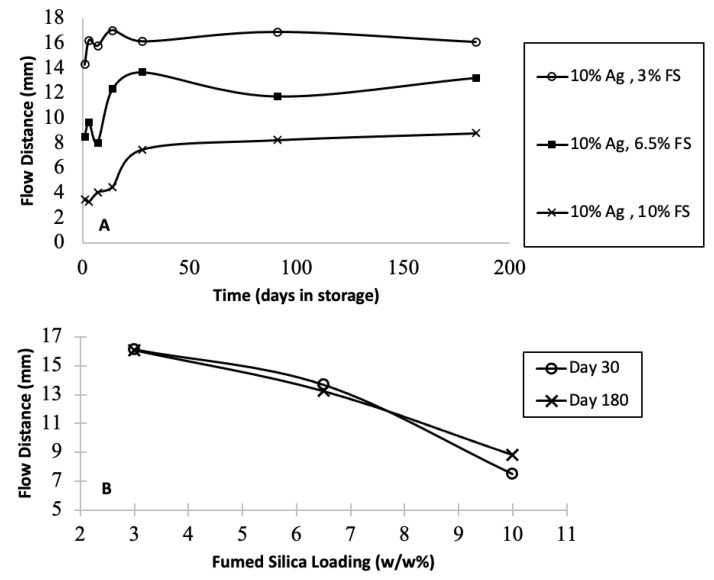
(**A**) Colloidal flow measurements for the resin loaded with 10 *w*/*w*% Ag ion releasing zeolites. with 3, 6.5, and 10 *w*/*w*% loadings of fumed silica in surgical guide resin as a function of time. Flow distances after 30 s were measured periodically over six months. (**B**) Flow measurements of the colloid loaded with 10 *w*/*w*% Ag-ion releasing zeolite as a function of fumed silica loading at 3, 6.5, and 10 *w*/*w*% in surgical guide resin. 30 s flow measurements were collected after one month and six months.

The antiviral colloid must remain stable for a specified shelf life when used in dental labs and clinics. The Ag-ion releasing zeolite colloid filler must not agglomerate and sediment prior to use in a 3D printer. In addition to visual inspection, flow distance measurements were utilized as a proxy for colloidal stability tests to determine whether the colloid could remain stable and flow at a predictable rate. Mixing fumed silica into the colloid is expected to prevent the colloid from phase separation during its intended shelf-life. Colloidal flow was measured as a function of time to determine how consistent the colloid flowed over the first six months of storage. If phase separation occurs, it is expected that the flow distances will significantly change as a function of time. Colloidal flow measurements are depicted in Figure 4. As seen in Figure 4A, there is an initial hysteresis in all 3 formulations over the first 2 weeks in storage before all 3 formulations held at a constant flow over the following five and a half months. As the fumed silica load increased, the flow distance of the colloid decreased. As seen in Figure 4B, all 3 formulations with different loading of fumed silica had a consistent flow between 1 and 6 months.

### 3.5. Viscosity

**Figure 5:**
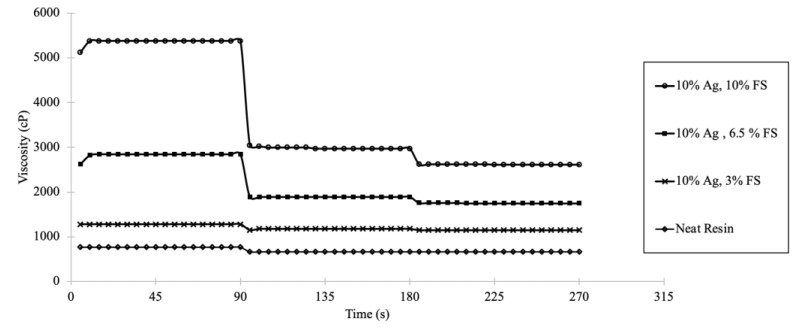
The viscosity of surgical guide resin and colloids with 10 *w*/*w*% Ag-ion zeolite with different loads of fumed silica (3, 6.5, and 10 *w*/*w*%) in surgical guide resin is reported as a function of time. The shear rate was increased at 90-s intervals from 1 rpm (0–90 s) to 10 rpm (91–180 s) to 25 rpm (181 to 270 s).

In addition to establishing the shelf life of the colloid, it is necessary to determine the viscosity of the colloids to assure that the resin is capable of continuous flow from the resin cartridge to the 3D printer. The colloid must flow into the printer tray at a targeted rate during active printing. If the addition of fillers drastically affects flow, it needs to be accounted for in the 3D printing process. Modification of the production process is necessary to account for the viscosity of the colloid relative to the neat resin. The viscosity of the neat surgical guide resin and 10 *w*/*w*% Ag zeolites with various loadings of fumed silica are represented in Figure 5. As seen in Figure 5, the viscosity increased for the colloid as the fumed silica loading increased. The neat resin and the colloid with 3.5 *w*/*w*% fumed silica were Newtonian as a function of time and were slightly pseudoplastic with minor decrease in viscosity with an increasing shear rate. The higher loading of fumed silica (6.5 and 10 *w*/*w*%) resulted in more pronounced pseudoplastic behavior as a function of increasing shear rate. Otherwise, the 6.5 and 10 *w*/*w*% fumed silica formulations appeared Newtonian as a function of time.

### 3.6. Flexural Strength

**Figure 6:**
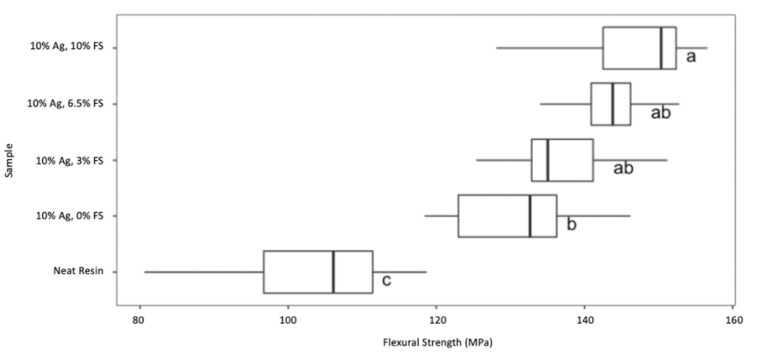
Flexural strength boxplot of composites with 10 *w*/*w*% Ag-ion zeolites with and without fumed silica. in surgical guide resin. An ANOVA test was completed and used in a pairwise comparison of means (Tukey’s test) at a 95% confidence interval to determine superscript groupings. Samples were sorted by the highest median value to the lowest median value.

Ultimately, the antiviral colloids prepared in this study are to be 3D printed and utilized as a dental material. It is crucial to understand the mechanical properties of the final composites. The flexural strength was determined for a polymer made from the neat resin and compared to composites made with Ag-ion releasing zeolites and different loadings of fumed silica. The flexural strength values, measured in megapascals (MPa), of each colloid are presented in a boxplot in Figure 6. A one-way ANOVA test was completed to determine that the data means are statistically significant, resulting in a *p*-value of less than 0.05. A multiple comparison of each mean (Tukey’s test) was then completed at a 95% confidence interval, and groupings are determined by each lettered superscript above. The letters denote groups that are not statistically different. For example, all bars with an “A” cannot be said to be statistically different from one another. However, the bar labeled “C” is statistically different from the rest of the composites since no other group is labeled “C”. The boxed area represents the interquartile range, formed by the 25th and 75th percentile observations in the group. The vertical line within the box is the median. The whiskers/horizontal lines represent the 1st and 4th quartiles, starting at the minimum for the left whisker and ending at the maximum for the right whisker.

The average value of each colloid, sorted by filler type, is listed in Table 1. The addition of Ag-ion releasing zeolites significantly increased the flexural strength of all materials compared to the unfilled resin. Further, the addition of the highest load of fumed silica (10 *w*/*w*%) increased the flexural strength of the zeolite filled composite relative to the zeolite filled composite formulated without fumed silica.

### 3.7. Silver Ion Release

**Figure 7:**
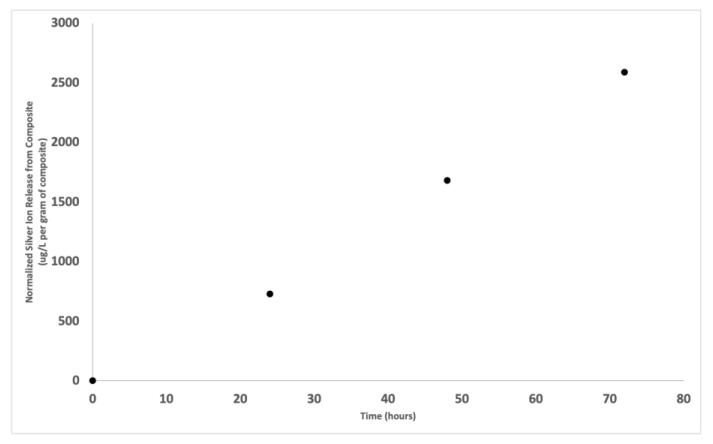
Normalized concentration of silver ions released from a composite loaded with 10 *w*/*w*%. silver ion releasing zeolite filler. The plot is the concentration of the silver ions in μg/L per gram of formulation as a function of time in hours.

The ion release profile of silver ions from the polymerized composite containing 10 *w*/*w*% Ag-ion releasing zeolites is shown in Figure 7. The graph depicts the μg/L of silver ions released per gram of composite as a function of time. The ultrapure water was tested for the presence of silver prior to composite submersion. This represents the 0 μg/L at time 0.

### 3.8. Antiviral Activity

**Figure 8:**
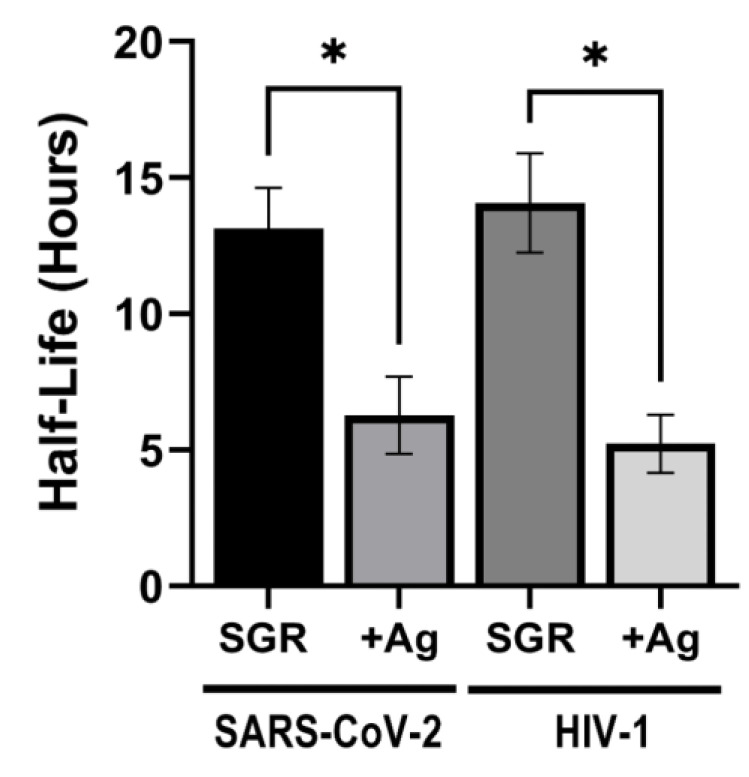
Antiviral activity of a composite loaded with 10 *w*/*w*% of a Ag-ion releasing zeolite. The average half-life of SARS-CoV-2 and HIV-1 incubated on the polymerized neat resin (SGR) or embedded with silver zeolite (+Ag). Data is the average of a minimum of three exposure experiments and * denotes a *p* < 0.05 by unpaired *t*-test.

The effectiveness of the Ag-ion releasing zeolite composite was tested for antiviral activity against SARS-CoV-2. For each experiment, six aliquots of virus were deposited on replicate composite surfaces and incubated in humidity chambers. Virus aliquots were recovered at 0, 4, 8, 24, 48, and 72 h and the amount of viable virus recovered quantified by plaque assays using Vero E6-A2T2 cells. The data for each exposure experiment was plotted over time and the half-life of the virus calculated from one phase exponential decay curves. Exposure experiments were performed a minimum of three times. As depicted in Figure 8, the average half-life of SARS-CoV-2 on the control SGR surface was 12.7 h. In contrast, the average half-life of SARS-CoV-2 exposed to SGR containing the Ag-ion zeolite composite was 6.7 h, a 1.9-fold reduction. To further test the extent of antiviral activity of the Ag-ion zeolite composite, we also tested its effect on the stability of human immunodeficiency virus type 1 (HIV-1). SARS-CoV-2 and HIV-1 are both an enveloped RNA virus. However, SARS-CoV-2 is a member of the *Retroviridae* family with different replication mechanisms. HIV-1 was applied to both the control neat SGR and the Ag-ion zeolite composite as for SARS-CoV-2, but the samples were titered on TZM-bl indicator cells to measure virus viability over time. Exposure of HIV-1 to the silver zeolite containing resin also resulted in a significant reduction in the average half-life of HIV-1 (Figure 7). The average half-life of HIV-1 on SGR was 14.1 h, which was reduced to 5.2 h on the SGR containing Ag-ion zeolite. Overall, this was a 2.7-fold reduction in the half-life of HIV-1.

## 4. Discussion

The purpose of this study was to incorporate Ag-ion releasing zeolites and fumed silica into 3D printable colloids to examine the possibility of creating a 3D printable colloid that results in a composite with antiviral properties. Fumed silica was added to slow down phase separation of the colloid. The inclusion of Ag-ion releasing zeolite fillers and fumed silica can result in a composite with acceptable mechanical properties. Our objective was to generate stable, antiviral colloids capable of predictable flow long after initial formulation while maintaining the mechanical properties of the final 3D printed part.

Appropriate mixing times and conditions were established to homogenously distribute the fillers, as depicted by the SEM image in Figure 1B. Initially, the photopolymerization kinetics of the neat resin was compared to the Ag-ion releasing zeolite containing colloid. As depicted in Figure 2, statistically the neat resin and the Ag-ion releasing zeolite loaded resin polymerized at comparable rates. Considering the resolution of a typical SLA printer is between 10 and 100 μm, conversion experiments obtained on an FTIR equipped with an ATR crystal should provide insight into how the filler effects the polymerization of the first 2 μm of the colloid in contact with the surface of the ATR crystal. The results suggest that at the percent conversion at this depth is not significantly affected by the presence of the Ag-ion releasing zeolite and that the presence of the silver loaded zeolite does not chemically inhibit polymerization.

While the zeolite does not inhibit polymerization, the presence of a filler will eventually reduce the depth of cure of the colloid relative to the neat resin. A challenge associated with all photopolymerized systems deals with the eventual limitations of how far the light can penetrate the sample to effectively convert the monomer to a polymer [11]. This challenge is greater yet when the resin system is filled with particles, especially with a mismatch of the refractive index between the filler and the continuous phase. Ultimately, there is a finite depth that the polymerization can occur. The resolution along the z-axis is determined by the choice of photoinitiator and the efficiency of the light source [1].

The guidelines set forth by ISO 4049 was used to determine the depth of cure in this study. As seen in Figure 3, the colloid loaded with 10 *w*/*w*% of Ag-ion releasing zeolite decreased the measured depth of cure. This is due to the effect the Ag zeolite has on the curing process. As shown in Figure 3, the 10 *w*/*w*% Ag releasing zeolite colloid resulted in a reduction of the depth of cure compared to the unfilled resin. The mismatch of refractive index between the Ag-ion releasing zeolite and the unfilled resin is the most likely explanation for this observation. The refractive index mismatch between the two dissimilar materials results in a bending of light that ultimately will reduce the depth that the cure light can penetrate into the material with enough energy to promote the reaction. Additionally, another factor for the observed reduction in depth of cure that needs to be considered is the competition between the filler particle and the photoinitiator for light absorption [111]. While the Ag-ion releasing zeolite filler produced a decrease in depth of cure, the cure depth recorded significantly exceeded the 10 to 100 μm resolution depth that is used in the layered photopolymerization of the 3D printer by 14 to 140 times. When considering the data collected from both the FTIR-ATR and depth of cure experiments, it seems that the inclusion of Ag-ion releasing zeolite fillers into a 3D printable resin with a depth of cure of 1400 μm in 5 s could allow for the printing of components with standard resolution of 10 to 100 μm in a comparable amount of time when compared to the neat resin.

Flow was measured as a function of storage time to determine the stability of the colloids. Over time, all fillers are expected to eventually flocculate and sediment after initial mixing in the continuous phase. Therefore, fumed silica was added to the colloid to slow down the flocculation and sedimentation of the antiviral fillers. Fumed silica imparts colloidal stability by electrostatic repulsion, and therefore, fumed silica should aid with the distribution of the zeolites and ultimately slow down the flocculation of the filler. Although the colloids were mixed homogenously, hysteresis is expected, resulting in varying flow measurements during the first month of data collection. After a period of hysteresis, the colloid is expected to exhibit a steady state of flow.

The flow of colloids with zeolite fillers and fumed silica are presented in Figure 4 to determine if the colloids are capable of remaining stable over the first six months. Each measurement was taken after 30 s of flow and presented as a function of time or filler loading. An initial flow test determined that neat surgical guide resin flowed 20.5 mm after 30 s. Figure 4A represents the flow distances of 10 *w*/*w*% Ag-ion releasing zeolites with 3, 6.5, and 10 *w*/*w*% loadings of fumed silica. These colloids underwent a period of hysteresis prior to the one-month flow measurement. The 10 *w*/*w*% Ag colloids featured longer flow distances with lower loadings of fumed silica and shorter flow distances with higher loadings of fumed silica. The decrease in flow is presumed to be due to the corresponding increase in viscosity generated by the incorporation of fumed silica. Variations in flow did not surpass 2 mm between each measurement post hysteresis. Figure 4B represents the flow distances of each colloid as a function of fumed silica loading after 30 days and after 180 days. Both plots show a decrease in flow distances with higher loadings of fumed silica.

Understanding the flow behavior of colloids is essential to determine its potential use in a 3D printer. A resin cartridge empties into a printer tray and must maintain its expected monomer level in the printer reservoir. Flow from the cartridge to the reservoir needs to be understood to maintain specific flow needed to print. After an initial two to four week period of hysteresis, the colloid remained stable and did not feature any drastic fluctuations in distances. This may suggest that the 10 *w*/*w*% Ag colloid loaded at either 3, 6.5 or 10 *w*/*w*% of fumed silica is capable of remaining stable over six months in a 3D printer resin cartridge.

Viscosity is the measure of friction within the fluid. The flow behavior of fluids can be separated into two categories: Newtonian and non-Newtonian. Newtonian fluids do not change viscosity in response to changes in stress (e.g., shear rate, time). Viscosities of non-Newtonian fluids are sensitive to change in stress and can be further classified as pseudoplastic or dilatant materials. Most dental materials depict pseudoplastic properties where the fluid decreases in viscosity when the shear rate is increased. The viscosity of fluid under constant shear stress can be constant over time (Newtonian) or change as a function of time (non-Newtonian). Non-Newtonian fluids can be classified as thixotropic or rheopectic as a function of time. A rheometer was utilized to determine the fluid properties of the antiviral colloids. Shear stress differences were achieved by mixing at 3 different speeds (1, 10, and 25 rpm). Each shear rate was held for 90 s to see the fluid behavior as a function of time. The obtained viscosity results were compared to the neat resin viscosity to see how the addition of antiviral fillers and fumed silica affects the viscosity of the colloid.

Viscosity measurements are shown in Figure 5. The neat resin has Newtonian properties as a function of time but has slight pseudoplastic properties as a function of shear rate when increased from 1 rpm to 10 rpm. The viscosity of the neat resin remains constant when the shear rate is increased from 10 rpm to 25 rpm. Each Ag-ion releasing zeolite colloid was non-Newtonian as a function of shear rate. The colloids viscosity did not change as a function of time when a constant shear rate was applied. Figure 5 shows that the viscosities decrease as shear rate increases, depicting pseudoplastic properties. Colloids with high loadings of fumed silica are more viscous than colloids with low loadings of fumed silica. Formulated colloids are more viscous than the neat resin and the formulations would need to be accounted for when implemented in 3D printing. The continuous flow from the cartridge to the printer must be increased to match targeted printing rates, or the printing time must be altered to match the flow of antiviral colloids coming out of the cartridge.

The antiviral colloids will ultimately be manufactured into dental materials using a 3D printer, and printed composites must withstand stress and maintain the minimum strength value of the original neat resin. Flexural strength was determined from three-point bend tests and were compared to the polymer made from neat resin. The added fillers must not reduce the strength of the initial neat resin composite to allow for optimal use of the material. The box and whiskers plot in Figure 6 and data reported in Table 1 was organized by the highest median value to the lowest median value to visualize the effect of fillers compared to the neat resin composite. The addition of Ag-ion releasing zeolites and fumed silica significantly increased the flexural strength relative to the neat resin. This could be due to the morphology of the zeolite particles. The zeolites are approximately 2.5 um in size with a very narrow particle size distribution. The inclusion of small sized particle fillers have been demonstrated to increase composite strength. Figure 7 demonstrates that silver ions are released from the polymerized composite. Presumably, the concentration of silver ions released from the material explain the origin of the antiviral activity of the composite.

In terms of antiviral activity, the results depicted in Figure 8 suggest the 3-D printed SGR composites containing Ag ion releasing zeolites decreased the half-life of SARS-CoV-2 by 47.24% and HIV-1 by 62.8%. This is consistent with previous work demonstrating that Ag is virucidal against HIV-1, HSV-1 vesicular stomatitis virus, SARS-CoV-1, and baculovirus [112]. The mechanism by which the Ag-ion releasing composite reduces SARS-CoV-2 and HIV-1 is incompletely understood. It has been proposed that Ag ions may interact with specific amino acids or RNA to inactivate virus [113,114,115]. As both SARS-CoV-2 and HIV-1 are enveloped viruses, it is likely Ag ions affect the viral envelope proteins or the membrane itself rather than viral RNA, capsid or nucleocapsid proteins [116]. Moreover, the availability of amino acids reactive to Ag may explain the differential impact of Ag on SARS-CoV-2 versus HIV-1 in our experiments. Interestingly, much recent work has focused on Ag nanoparticles, showing that Ag nanoparticles reduce the infectivity of HIV-1 and HSV-1 by direct binding to virions and blocking of viral binding and entry into the host cell [115,116,117,118,119].

## 5. Conclusions

The potential to stabilize antimicrobial fillers in a colloid using fumed silica for use in a 3D printer without degradation of mechanical properties has been demonstrated. Future studies are needed to explore the specific mechanisms by which Ag ions release from zeolites are virucidal. Additionally, further surface characterization of the printed parts are needed to provide insight for antiviral behavior. This may lead to improved methods to utilize Ag for virus elimination. Inclusion of zeolites have given preliminary results that suggest an improvement in mechanical behavior compared to the neat resin. Further mechanical testing, including compression and stress relaxation tests will be examined in the future. The 3D printed composites with Ag ions tend to brown in color over time. If this material were used for appliances where aesthetic concerns were prevalent, other stabilizers or colorants would need to be added to the colloid to address these requirements.

## Figures and Tables

**Table 1:** Average flexural strength of 10 *w*/*w*% Ag only zeolites with and without fumed silica in surgical guide resin.

Sample	Flexural Strength (MPa)
Neat Resin	102.42 ± 13.67
10 *w*/*w*% Ag Only/0 *w*/*w*% FS	130.83 ± 9.55
10 *w*/*w*% Ag Only/3 *w*/*w*% FS	137.28 ± 8.67
10 *w*/*w*% Ag Only/6.5 *w*/*w*% FS	143.33 ± 5.36
10 *w*/*w*% Ag Only/10 *w*/*w*% FS	146.25 ± 9.67

## Data Availability

Not applicable.
